# Revealing the Action Mechanism of Exogenous Hydrogen Sulfide Intervention in Colorectal Cancer Pathogenesis Based on Multiomics Analysis and Experimental Validation

**DOI:** 10.1155/humu/5595505

**Published:** 2026-01-23

**Authors:** Facai Cui, Chen Yu, Weifeng Zhao, Juan Shi, Yang Liu, Fengzhen Liu

**Affiliations:** ^1^ Department of Clinical Laboratory, Henan Provincial People′s Hospital, Zhengzhou, Henan, China, hnsrmyy.net; ^2^ Department of Pathology, Henan Cancer Hospital, Affiliated Cancer Hospital of Zhengzhou University, Zhengzhou, Henan, China, zzu.edu.cn; ^3^ Department of Oncology Laboratory, Henan Provincial People′s Hospital, Zhengzhou, Henan, China, hnsrmyy.net

**Keywords:** colorectal cancer, hydrogen sulfide, multiomics analysis, pathogenesis risk, therapeutic mechanism

## Abstract

Colorectal cancer (CRC) ranks among the leading causes of cancer‐related mortality worldwide. Hydrogen sulfide (H_2_S) has been found to possess a characteristic of anticancer, which may offer a potential novel treatment for CRC. Here, we discover the potential targets and mechanism of H_2_S intervention in CRC employing multiomics analysis and experimental validation. The key targets of H_2_S intervention in CRC were identified by integrating differentially expressed genes (DEGs) from tumor and normal tissues, the CRC‐associated genes, and the targets of H_2_S. The STRING and Cytoscape tools were explored to obtain hub genes. Functional enrichment analysis, assessment of diagnostic and prognostic significance, single‐cell datasets, and cell experiments were used to explore the impact of core targets on CRC and the potential mechanism through which H_2_S exerts regulatory effects on CRC. Our results identified 9250 genes closely linked to CRC from DEGs and CRC‐associated genes, 505 targets for H_2_S, and 322 potential targets of H_2_S intervention in CRC. Subsequently, five hub genes were filtered, including MAPK1, MAPK3, JUN, ESR1, and AKT1. The 322 common targets were enriched in the cellular stress responses and IL‐17 signaling pathway. Additionally, MAPK3 had good diagnostic and prognostic value for CRC. JUN was highly expressed in immune cells. Cell experiments showed that sodium hydrosulfide (NaHS), a donor of H_2_S, prominently inhibited cell proliferation, promoted cell apoptosis for CRC, and downregulated the expression of MAPK1, MAPK3, AKT1, and JUN. Taken together, this study elucidates the possible genes and therapeutic mechanisms underlying exogenous H_2_S intervention in CRC, thereby laying a foundation for the further development of H_2_S‐based therapeutic strategies in CRC management.

## 1. Introduction

Colorectal cancer (CRC) is the third most commonly diagnosed cancer worldwide [1]. In 2024, CRC accounted for 7.6% of all new diagnoses and ranks second in cancer deaths [2]. CRC research has focused on early diagnosis, therapeutic targets, and treatment methods [3]. Currently, the primary therapeutic approaches for CRC encompass surgical intervention, chemotherapy, radiation therapy, targeted treatment, and immunotherapy [4, 5]. Despite the ability of these treatment modalities to extend patient survival, disease recurrence and metastasis continue to be the primary causes of therapeutic failure [6]. Therefore, finding safe and effective treatment methods remains the current focus of CRC treatment.

Hydrogen sulfide (H_2_S), as a gas, exhibits diverse functions in the occurrence and development of various diseases [7]. H_2_S can not only play a protective role but also may be involved in the pathological process of diseases [8, 9]. Meanwhile, H_2_S can regulate apoptosis and autophagy, thereby influencing the survival and death of cells [10, 11]. Furthermore, the effects of H_2_S depend on its concentration, the target tissue, and the specific disease environment [8]. Current research has found that H_2_S is involved in multiple stages of CRC initiation and progression, with its underlying mechanisms and regulatory factors becoming a focus of widespread research interest [7, 12]. However, how exogenous H_2_S affects CRC progression by regulating specific molecular targets is still unclear. The application of H_2_S in biological experiments is limited due to its volatility, instability, and toxicity [13–15]. Current research indicates that sodium hydrosulfide (NaHS) is a common donor of H_2_S, and it has potential antitumor effects in various cancer treatments [16, 17]. Therefore, due to the characteristics of easy quantification and safety, NaHS is widely used in biological experiments as an agent to evaluate the effects of H_2_S [18, 19].

With the development of medical technology, omics technology is crucial for analyzing the pathogenesis of diseases [20]. However, a single omics technique has limitations in elucidating disease mechanisms. Currently, the multiomics approach has significant advantages in cancer research in modern medicine, such as comprehensive molecular characteristics analysis, accurate disease classification, effective biomarker discovery, and in‐depth drug target identification [21]. For example, the comparison of multiomics data between healthy and diseased individuals can identify biomolecules involved in the occurrence and development of tumors, which may serve as drug targets for the exploitation of new therapeutic agents [22]. Integrating multiomics data analysis can identify the molecular characteristics related to tumor immunity in CRC, thereby providing more precise targets for immunotherapy [23]. Therefore, the multiomics approach can reveal the complex mechanisms of cancer occurrence, development, and metastasis by integrating genomics, transcriptomics, proteomics, metabolomics, and other multilevel datasets, thereby providing new strategies for cancer prevention, diagnosis, and treatment [24, 25].

**Figure 1:**
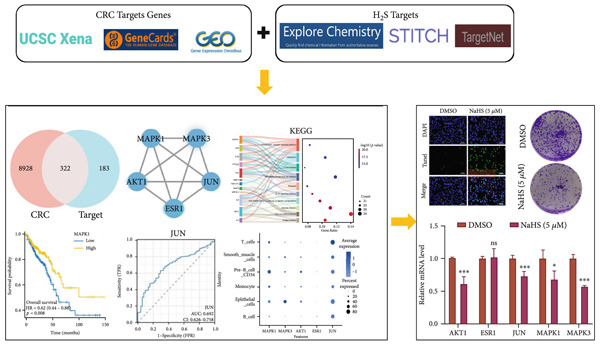
Workflow of the study.

Our study is aimed at systematically exploring the physiological mechanism of exogenous H_2_S in treating CRC through the combination of multiomics analysis and cell experiments. Here, we constructed an integrated network of H_2_S‐related targets and CRC‐associated genes to identify key targets of H_2_S intervention in CRC and the interaction patterns between molecules. Then, we explored the diagnostic and prognostic significance of core genes and verified the distribution of core genes in the tumor microenvironment for CRC. Finally, we evaluated the therapeutic effects of exogenous H_2_S in CRC cells, providing experimental support for its potential use as a novel drug in CRC therapy. The study workflow is shown in Figure 1.

## 2. Materials and Methods

### 2.1. Acquiring CRC‐Associated Databases

The mRNA data and matched clinical data of The Cancer Genome Atlas (TCGA) cohort, comprising 473 tumor tissues and 41 normal tissues, were sourced via the UCSC Xena (http://xena.ucsc.edu). GSE156355, GSE234804, and GSE277669 were downloaded from the Gene Expression Omnibus (GEO) (https://www.ncbi.nlm.nih/). GSE156355 contained RNA sequencing data with six tumor tissues and six normal tissues. For single‐cell RNA sequencing data, GSE234804 included three primary CRC patients and GSE277669 included 1 CRC patient. Additionally, GeneCards (https://www.genecards.org) and TCGA databases offered CRC‐related targets.

### 2.2. Screening H_2_S‐Associated Targets

According to the screening criteria of Yu et al. [26], H_2_S‐associated targets were sourced from the STITCH (http://stitch.embl.de/), TargetNet (http://targetnet.scbdd.com), and PubChem (https://pubchem.ncbi.nlm.nih.gov) databases. Briefly, the “CAS 7783‐06‐4” was queried in the PubChem database to acquire the “2D.sdf” file, and the file was submitted to ChEMBL (https://www.ebi.ac.uk/chembl/) to acquire the SMILES notation for H_2_S. Subsequently, the TargetNet database [27] and STITCH database [28] were utilized to predict more pharmacological targets by searching for the SMILES number of H_2_S. Additionally, the PubChem database was used to search “CAS 7783‐06‐4” to obtain more potential targets [29]. The above‐acquired targets were standardized through the UniProt database (https://www.uniprot.org/) by applying “Homo sapiens” as the species filter. Finally, we combined the targets obtained from the three databases and removed duplicate targets to acquire the H_2_S‐associated targets.

### 2.3. Acquisition of Differentially Expressed Genes (DEGs)

For dimensionality reduction and feature extraction within TCGA and GSE156355 datasets, principal component analysis (PCA) was conducted utilizing the “factoextra” in R software. Then, TCGA and GSE156355 databases were explored for differential gene analysis using the limma and DESeq2 R package, according to the parameter with *p* value less than 0.05 and |log_2_FoldChange| greater than 1. Heatmap and volcano plot were employed for visualizing DEGs using the “ggplot2” in R software.

### 2.4. Creation of Protein–Protein Interaction (PPI) Network and Screening Hub Gene

We integrated DGEs and CRC‐related genes and deleted duplicate genes to obtain the genes most closely associated with CRC. Then, a Venny diagram was utilized to obtain the common targets between the genes most closely associated with CRC and H_2_S‐associated targets. A PPI network was built using the STRING database (https://cn.string-db.org/) to predict interactions among the shared targets. Cytoscape software 3.9.1 was employed for visualizing and refining the PPI network and computing different parameters for each node. Then, the CytoHubba plugin (Version 0.1) in Cytoscape 3.9.1 was used to screen the Top 15 core targets from betweenness centrality, closeness centrality, and degree (number of connections), respectively. The Venny diagram showed the overlapping genes between the above three networks. The MCODE plugin in Cytoscape 3.9.1 was performed to verify the hub genes obtained from PPI protein clustering analysis.

### 2.5. Prediction of the Biological Functions

The “clusterProfiler” in R package was employed for conducting Gene Ontology (GO) annotation on shared targets from H_2_S‐treated CRC. The biological process (BP), cellular component (CC), and molecular function (MF) categories were selected, and statistically significant annotations (*p* values < 0.05) were included for further analysis. Meantime, Kyoto Encyclopedia of Genes and Genomes (KEGG) pathway enrichment analysis was performed by running the “ggplot2” in R package.

### 2.6. Diagnostic Value Analysis and Prognostic Evaluation

TCGA cohort was explored for receiver operating characteristic (ROC) curve creation and Kaplan–Meier survival analysis. The area under the ROC curve (AUC) was calculated to evaluate the diagnostic value of core genes. The “pROC” package in R software was used to establish the ROC curve, and an AUC value > 0.80 was considered a good diagnostic effect [30]. Kaplan–Meier survival analysis was constructed based on overall survival (OS) and progression‐free survival (PFS) of CRC patients. The “survival” and “survminer” packages in R were applied to perform Kaplan–Meier survival analysis.

### 2.7. Single‐Cell RNA Sequencing Data Analysis of Hub Genes in CRC

Single‐cell RNA sequencing data analysis data was read from GSE234804 and GSE277669 by the “Seurat” R package. Data were processed with reference to a previous study [31]. Briefly, cells were selected according to the following criteria: cells (containing > 300 genes), cells (expressing < 7000 genes), and cells (containing < 1% of mitochondrial genes). The Top 2000 highly variable genes were selected through the use of FindVariableFeatures and NormalizeData functions, and all samples were followed by PCA. The uniform manifold approximation and projection (UMAP) dimensionality reduction technique showed clustered cells. In addition, the “SingleR” package was applied to annotate the cell type, and the “DotPlot” function in Seurat showed the expression pattern of each hub gene among clusters.

### 2.8. Culture and Treatments of Cell Lines

Based on the recent research protocols, we conducted culture experiments using two CRC cell lines, HT‐29 (species: human; sex: female; tissue of origin: a primary tumor sample was isolated from a 44‐year‐old white female patient with colon cancer; RRID: CVCL_0320) and HCT‐116 (species: human; sex: male; tissue of origin: derived from the lymph nodes of a 51‐year‐old male patient with colon cancer; RRID: CVCL_0291) [32]. The Type Culture Collection of the Chinese Academy of Sciences (Shanghai, China) provided HT‐29 and HCT‐116 cell lines in February 2025. HT‐29 and HCT‐116 cells have not been previously reported as misidentified or contaminated. The cells were authenticated through short tandem repeat (STR) profiling. The matching rate of cells (HT‐29 and HCT‐116) subjected to STR testing was 100%. No cross‐contamination was identified following mycoplasma testing. Cells were cultured in DMEM (Gibco, Grand Island, NY, United States) supplemented with 10% (*v*/*v*) heat‐inactivated FBS (Thermo Fisher Scientific, Waltham, MA, United States) at 37°C under a 5% CO_2_ atmosphere. Based on the previous studies, NaHS can be utilized to construct various biological and medical models for investigating the physiological and pathological effects of H_2_S [16, 18]. Therefore, HT‐29 and HCT‐116 cell lines were incubated with NaHS (TargetMol Chemicals Inc., Boston, MA, United States) for 24 h. In this experiment, NaHS was diluted with DMSO and added to the culture medium.

### 2.9. Cell Counting Kit‐8 (CCK‐8) Assay

The CCK‐8 kit from Beyotime Biotechnology (Shanghai, China) was employed for measuring cell viability, following the manufacturer′s guidelines. We dispensed 100 *μ*L of culture medium into each well of the 96‐well plate. Then, after cells were treated with 0.1–100 *μ*M of NaHS for 24 h, 10 *μ*L CCK‐8 solution was added into each well. After incubation for 1 h, the absorbance (OD value) of cells was measured at 450 nm. By calculating the IC_50_ values, HT‐29 and HCT‐116 cells were treated with 5 and 10 *μ*M NaHS for 24 h, respectively, for subsequent experiments.

### 2.10. Terminal Deoxynucleotidyl Transferase–Mediated dUTP Nick‐End Labeling (TUNEL) Staining

To investigate the effect of NaHS on the apoptosis of HT‐29 and HCT‐116 cells, we used the TUNEL staining method to detect cell apoptosis by the One Step TUNEL Apoptosis Assay Kit (green fluorescence, Beyotime Biotech). Specifically, after 24 h NaHS treatment, HT‐29 and HCT‐116 cells were fixed with 4% paraformaldehyde for 30 min and permeabilized with 0.3% Triton X‐100 in PBS for 5 min at room temperature. Next, 50 *μ*L of the biotin‐labeled solution was added to the sample, incubating for 60 min at 37°C in the dark. Finally, the samples were sealed with antifade mounting medium (Beyotime Biotech) and visualized under a fluorescence microscope.

### 2.11. Colony Formation Assay

Briefly, cells (1 × 10^3^) were plated in a six‐well plate and cultured in DMEM with 10% FBS for 14 days. After the samples were washed three times with PBS, 4% paraformaldehyde was employed for fixing cells for 20 min. Crystal violet (Beyotime Biotech) was employed for staining cells for 15 min, and cells were visualized under light microscopy.

### 2.12. Quantitative PCR (q‐PCR)

Following the TRIzol reagent (Thermo Fisher Scientific) protocol, total RNA was extracted from cells. The isolated RNA was synthesized into cDNA by utilizing the TaKaRa (Tokyo, Japan) kit. Finally, gene expression was assessed via q‐PCR with the 2× ChamQ SYBR qPCR Master Mix (Vazyme, Nanjing, Jiangsu, China), employing gene‐specific primers (Table 1). GAPDH served as an internal reference. The 2^–ΔΔCt^ method determined relative quantification.

**Table 1:** Primer information for the genes.

**Genes**	**Forward primer (5** ^′^ **-3** ^′^ **)**	**Reverse primer (5** ^′^ **-3** ^′^ **)**
AKT1	CTGGGCAAGGGCACTTTCGG	AGGCGGTCGTGGGTCTGGAA
ESR1	ATGGAGTCTGGTCCTGTGAGG	GCTGTTCTTCTTAGAGCGTTTGA
JUN	TACGCAAACCTCAGCAACTTCAA	ATCCGCTCCTGGGACTCCAT
MAPK1	TTCCCAAATGCTGACTCCAA	TCGGGTCGTAATACTGCTCC
MAPK3	TCAACACCACCTGCGACCTT	GCGTAGCCACATACTCCGTCA
GAPDH	GAAATCCCATCACCATCTTCC	TGAGTCCTTCCACGATACCAA

Abbreviations: AKT1, AKT serine/threonine kinase 1; AP‐1, transcription factor subunit; ESR1, estrogen receptor 1; GAPDH, glyceraldehyde‐3‐phosphate dehydrogenase; JUN, JUN proto‐oncogene; MAPK, mitogen‐activated protein kinase.

### 2.13. Statistical Analysis

Data were displayed in terms of means ± standard deviation (SD) from three independent experiments. SPSS 18.0 and GraphPad Prism 8.0 were explored for statistical analysis. The differences between the two groups were analyzed using Student′s *t*‐test. *p* value < 0.05 was considered significant.

## 3. Results

### 3.1. Identification of DEGs in CRC

Here, we initially used TCGA and GSE156355 cohorts to obtain DEGs in CRC. The PCA results of the two databases indicated that the DEGs discriminated CRC and normal tissues into two significantly distinct groups (Figure 2a,d,d). The identified DEGs in TCGA and GSE156355 were 1333 and 6557, respectively (Figures 2b, 2c, 2e, and 2f). Subsequently, we integrated DEGs and CRC‐related targets and removed duplicate targets to obtain 9250 targets closely associated with CRC. Meanwhile, we consolidated the target information of H_2_S from the STITCH, TargetNet, and PubChem databases, forming an H_2_S dataset with 505 targets.

Figure 2DEGs of the CRC obtained from TCGA and GSE156355 cohorts. (a) PCA plot showed the distribution of DEGs in normal and tumor groups in TCGA cohort. (b) Heatmap and (c) volcano plot of the 1333 DEGs in TCGA cohort. (d) PCA plot showed the distribution of DEGs in normal and tumor groups in the GSE156355 cohort. (e) Heatmap and (f) volcano plot of the 6557 DEGs in the GSE156355 cohort. Red color indicated upregulated genes, blue indicated downregulated genes, and the grey indicated genes with no significant changes.

**Figure:**
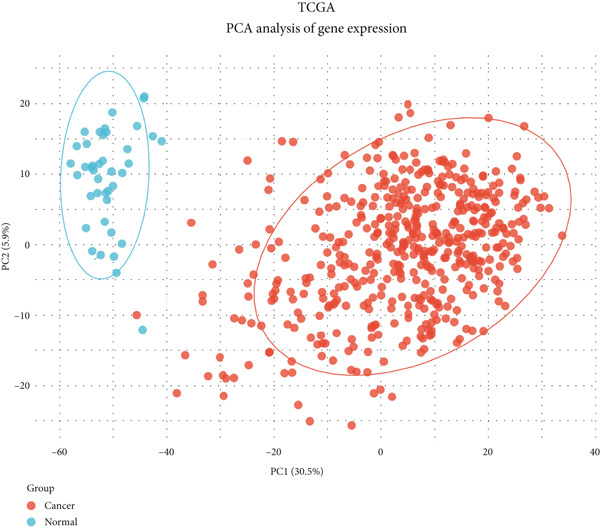
(a)

**Figure:**
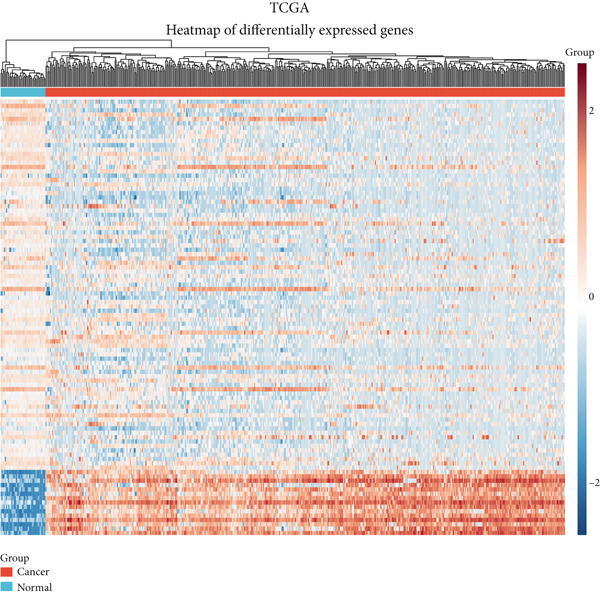
(b)

**Figure:**
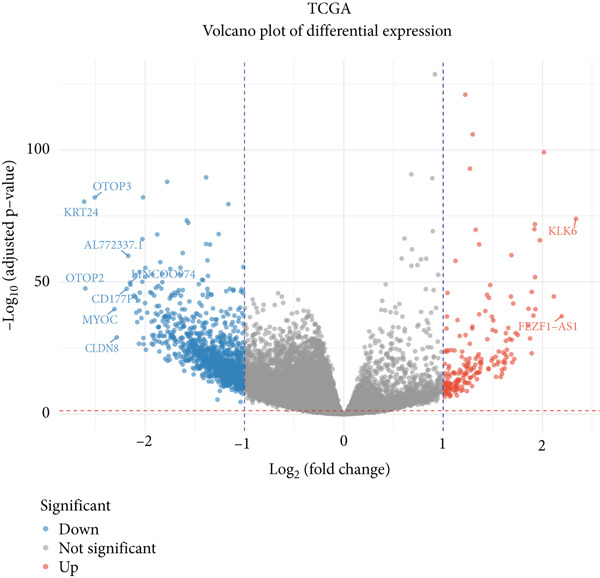
(c)

**Figure:**
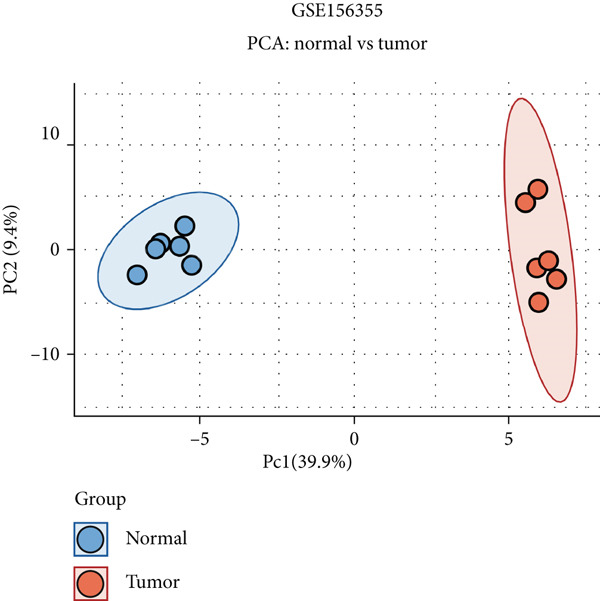
(d)

**Figure:**
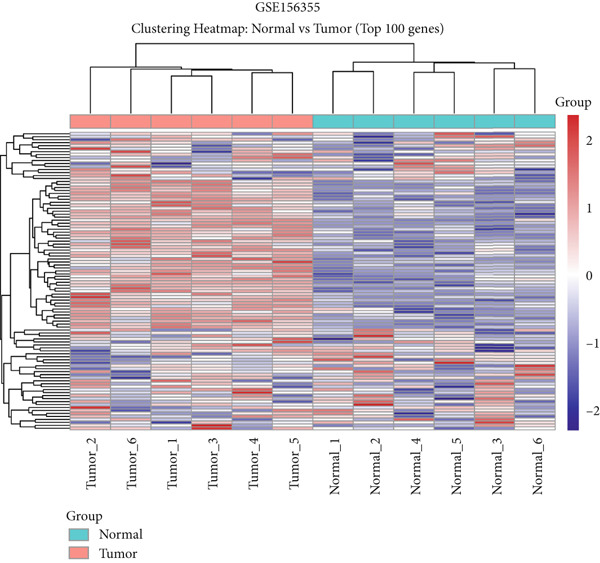
(e)

**Figure:**
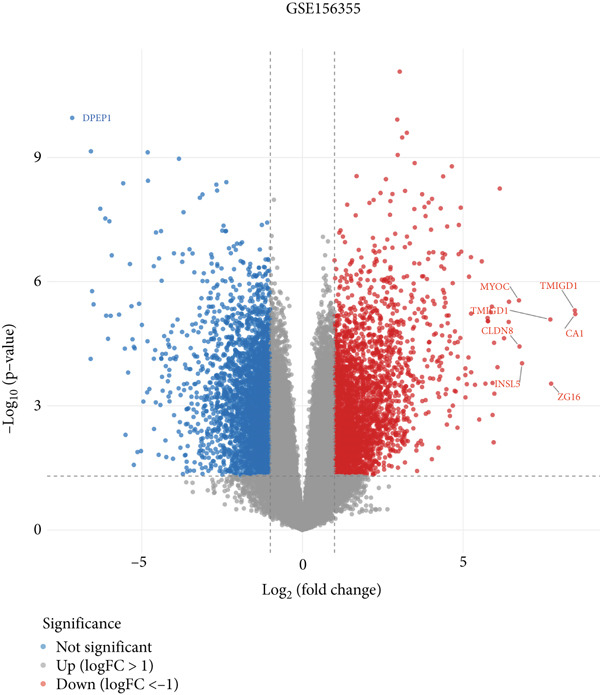
(f)

### 3.2. Acquisition of Hub Genes

To identify the potential targets underlying H_2_S intervention in CRC, we initially intersected the targets closely associated with CRC and the targets of H_2_S, obtaining 322 intersection genes (Figure 3a). Subsequently, Cytoscape software has visualized and refined the PPI network consisting of the Top 115 genes in degree values (Figure 3b). Figure 3c showed the significance and potential role of the nine core genes in the interaction network. Moreover, the cytoHubba plugin (Version 0.1) within the Cytoscape software with three key metrics—degree (Figure 3d), closeness centrality (Figure 3e), and betweenness centrality (Figure 3f)—has screened out the Top 15 targets from each network. Next, a Venny diagram has identified nine common hub genes in the above three networks (Figure 3g). Additionally, we utilized the MCODE plugin to identify critical hub gene modules (Figure 3h,i). Finally, we have identified five hub genes with the highest degree value: MAPK1, MAPK3, JUN, AKT1, and ESR1. This finding suggests their potential importance in the H_2_S regulation of CRC.

Figure 3Construction of PPI network and screening hub genes. (a) Venny diagram of common genes between targets closely associated with CRC and targets of H_2_S. (b) The PPI network of the Top 115 genes in degree values. (c) Core target network. (d) Network of Top 15 based on degree analysis. (e) Network of Top 15 based on closeness centrality analysis. (f) Network of Top 15 based on betweenness centrality analysis. (g) Venny diagram of common genes among the above three networks. (h) Network of the first MCODE plugin analysis. (i) Network of the second MCODE plugin analysis.

**Figure:**
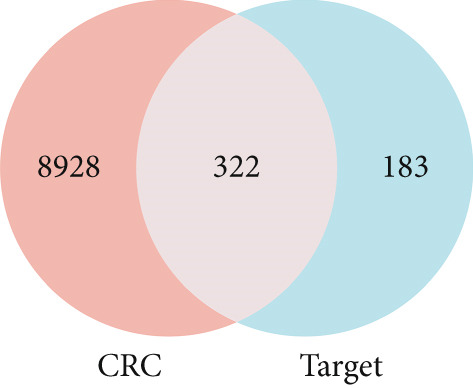
(a)

**Figure:**
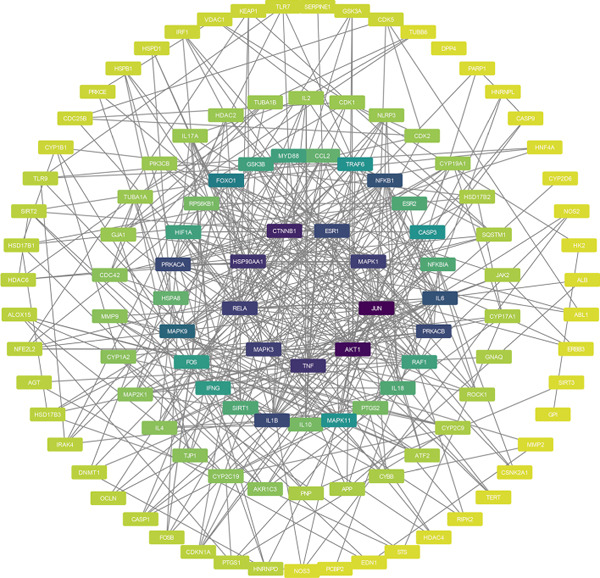
(b)

**Figure:**
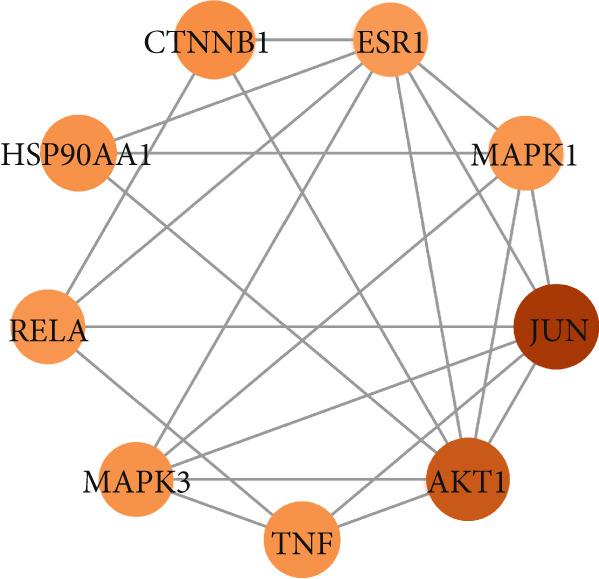
(c)

**Figure:**
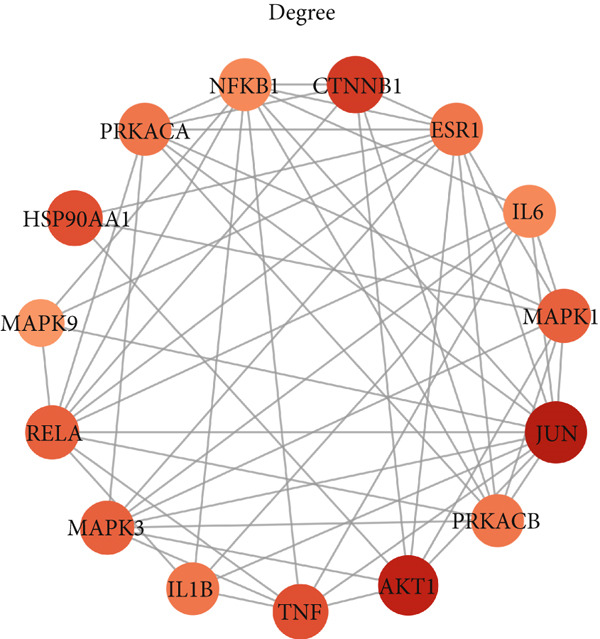
(d)

**Figure:**
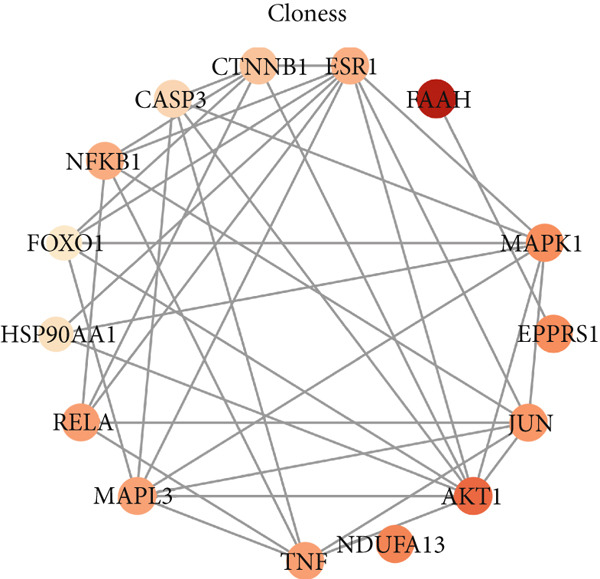
(e)

**Figure:**
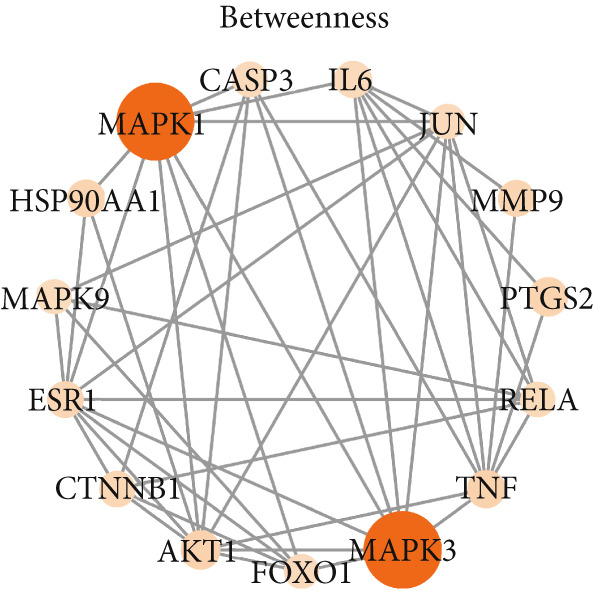
(f)

**Figure:**
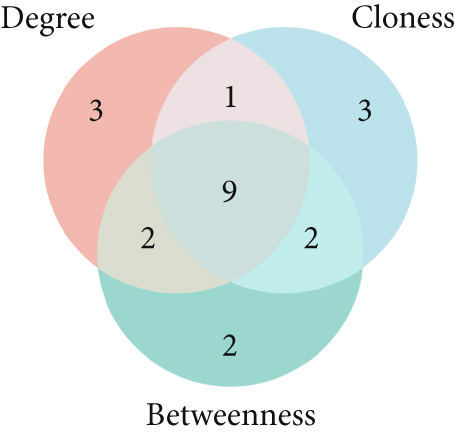
(g)

**Figure:**
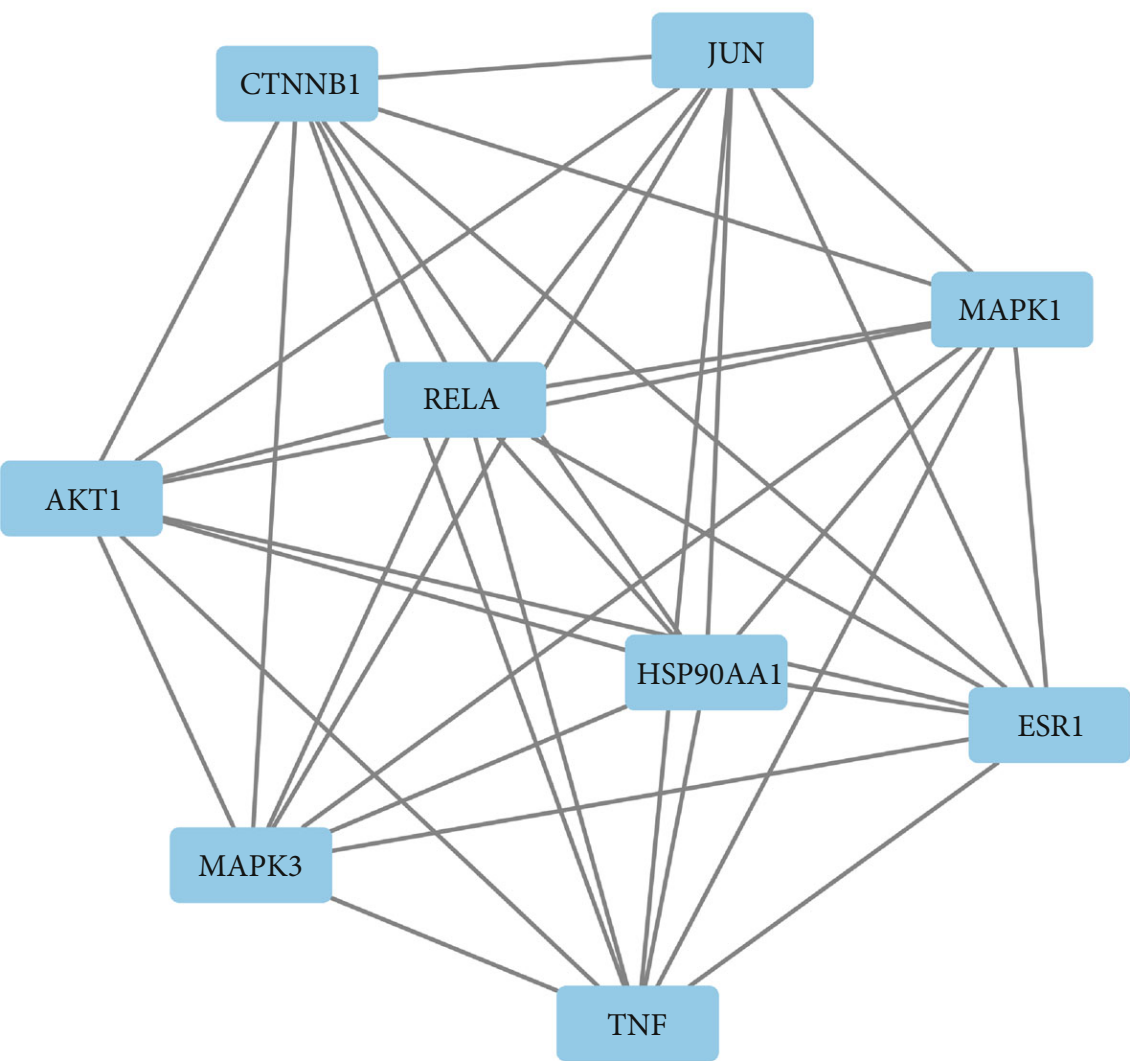
(h)

**Figure:**
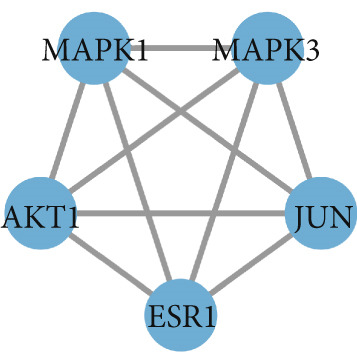
(i)

### 3.3. Prediction of the Biological Functions of Intersecting Genes

The 322 shared genes showed significant enrichment in various BP categories, such as aging, cellular response to biotic stimulus, response to lipopolysaccharide, response to oxygen levels, response to reactive oxygen species, and response to xenobiotic stimulus (Figure 4a). The coexpressed genes were enriched in several key CC: caveola, cytoplasmic vesicle lumen, ficolin‐1 rich granule lumen, focal adhesion, membrane microdomain, membrane raft, plasma membrane raft, postsynaptic membrane, secretory granule lumen, and vesicle lumen (Figure 4b). The cross genes were found to be involved in MF: carbonate dehydratase activity, heme binding, ligand‐activated transcription factor activity, protein serine kinase activity, tetrapyrrole binding, and so on (Figure 4c). The KEGG pathway analysis indicated that these cross genes were enriched in some pathways, such as IL‐17 signaling pathway, Chagas disease, and C‐type lectin receptor signaling pathway (Figure 4d). Meanwhile, we constructed a Sankey diagram, which clearly describes the interrelationships between the targets and the pathways (Figure 4e).

Figure 4GO and KEGG enrichment analyses of 322 potential targets of H_2_S intervention in CRC. (a) Targets of H_2_S‐treated CRC enriched in the Top 10 BP categories. (b) Targets of H_2_S‐treated CRC enriched in the Top 10 CC categories. (c) Targets of H_2_S‐treated CRC enriched in the Top 10 MF categories. (d) Targets of H_2_S‐treated CRC enriched in the Top 10 pathways. (e) Sankey diagram connecting the Top 10 pathways to the relevant targets.

**Figure:**
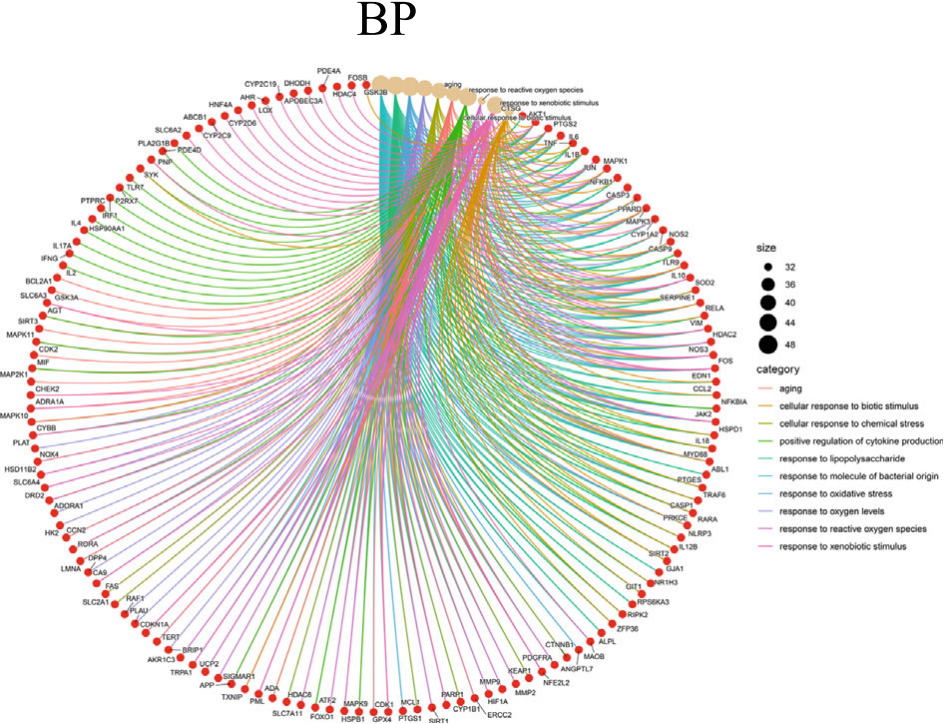
(a)

**Figure:**
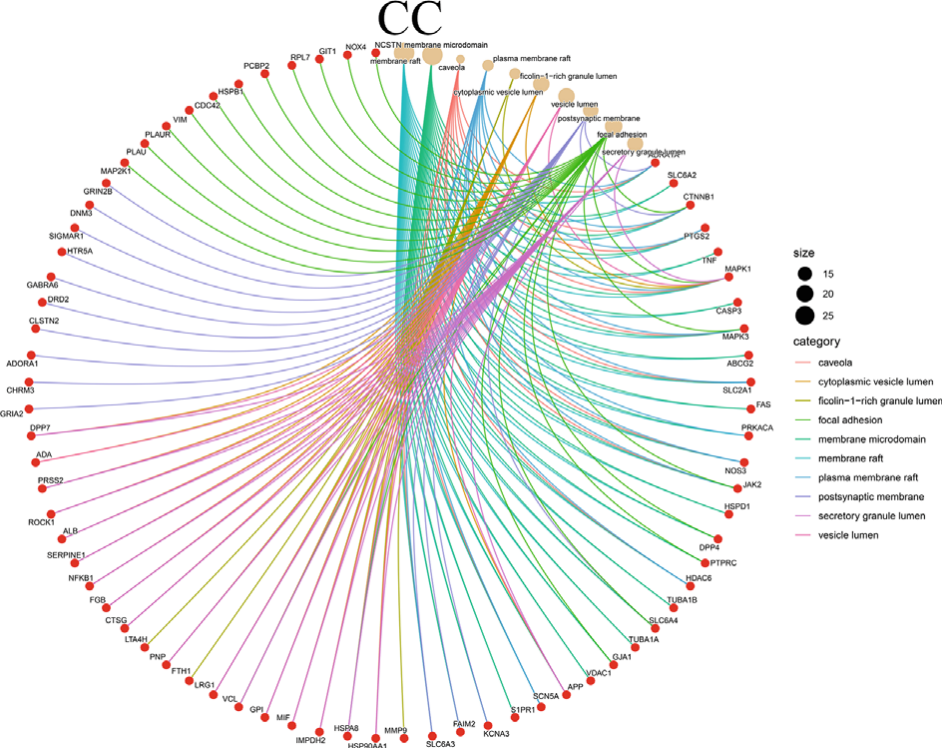
(b)

**Figure:**
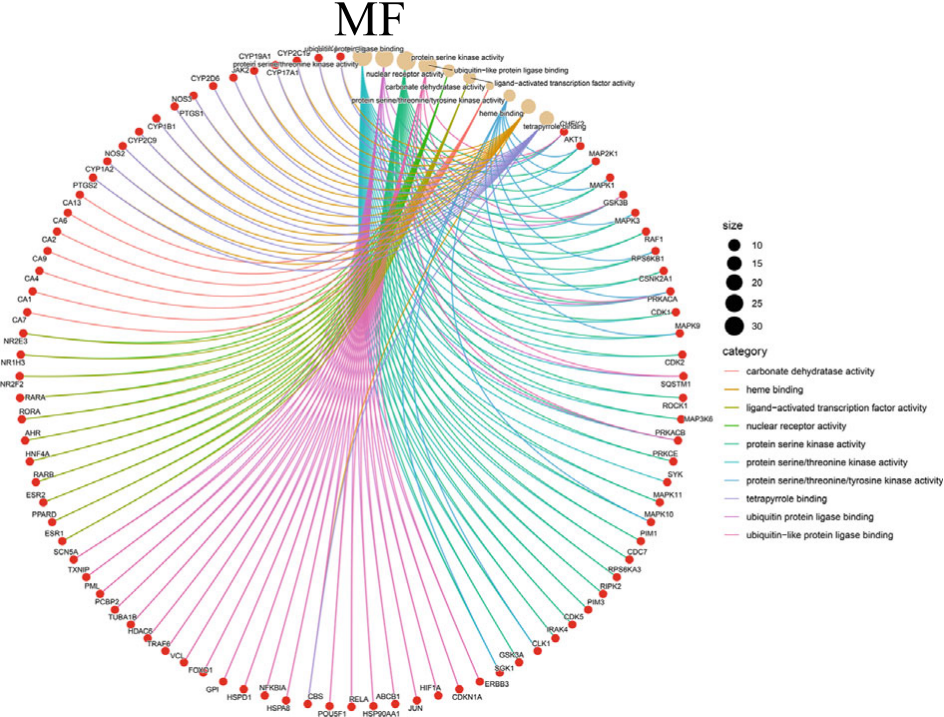
(c)

**Figure:**
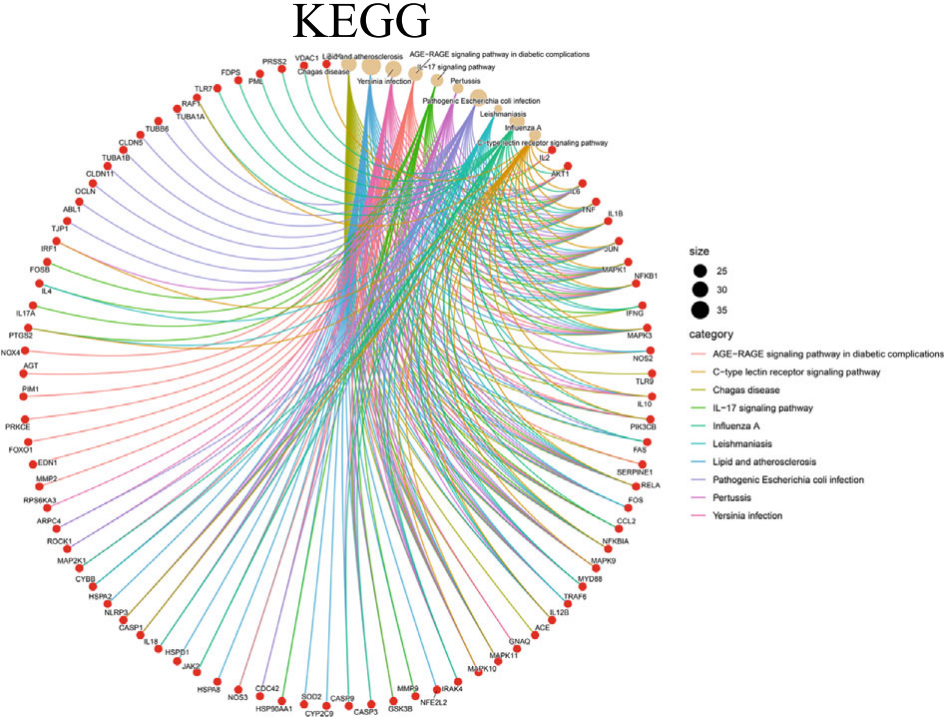
(d)

**Figure:**
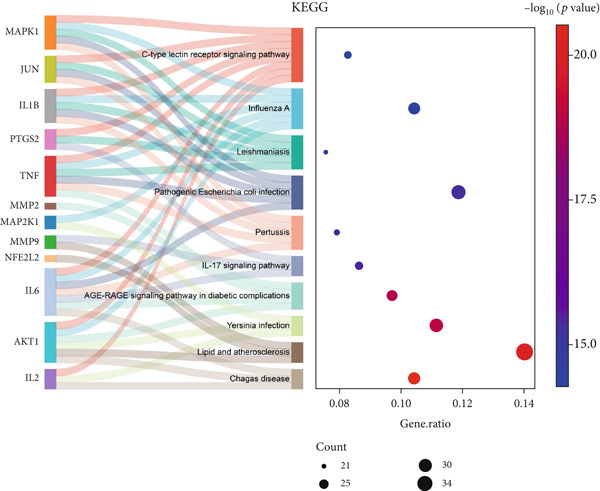
(e)

### 3.4. The Diagnostic Performance of Hub Genes

The AUC of AKT1 was 0.623 (CI: 0.562–0.684, Figure 5a), and the AUC of ESR1 was 0.848 (CI: 0.810–0.886, Figure 5b). Moreover, the AUC values of JUN, MAPK1, and MAPK3 were 0.692 (CI: 0.626–0.758, Figure 5c), 0.612 (CI: 0.556–0.669, Figure 5d), and 0.875 (CI: 0.836–0.914, Figure 5e), respectively. Taken together, the diagnostic performance of ESR1 and MAPK3 performed markedly better for CRC.

Figure 5Diagnostic value of five hub genes in TCGA cohort. (a) ROC curve of AKT1. (b) ROC curve of ESR1. (c) ROC curve of JUN. (d) ROC curve of MAPK1. (e) ROC curve of MAPK3.

**Figure:**
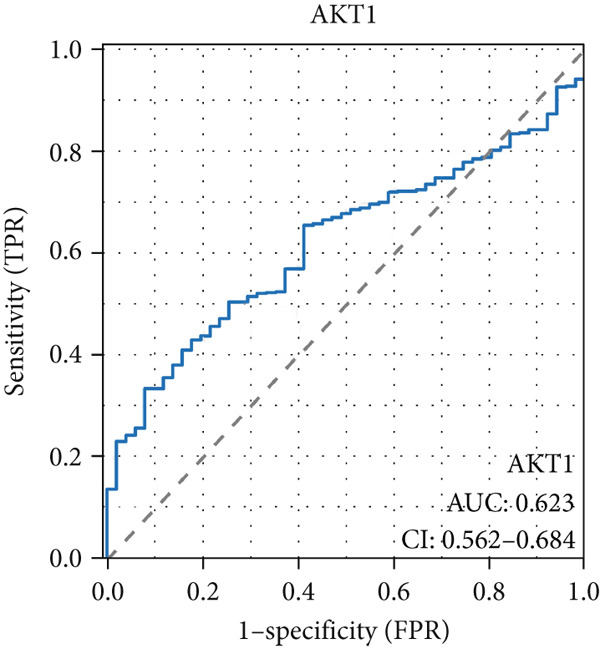
(a)

**Figure:**
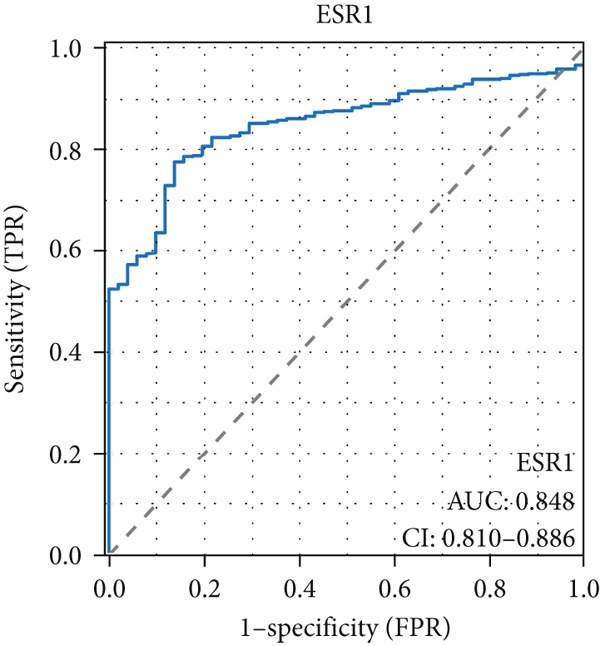
(b)

**Figure:**
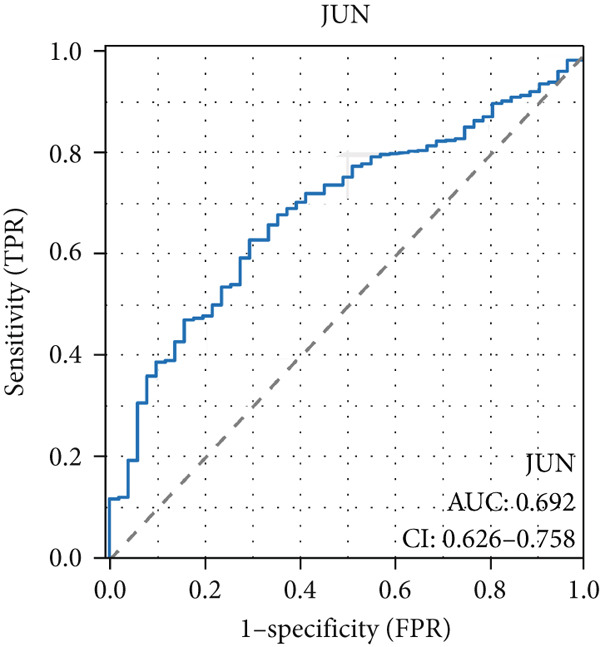
(c)

**Figure:**
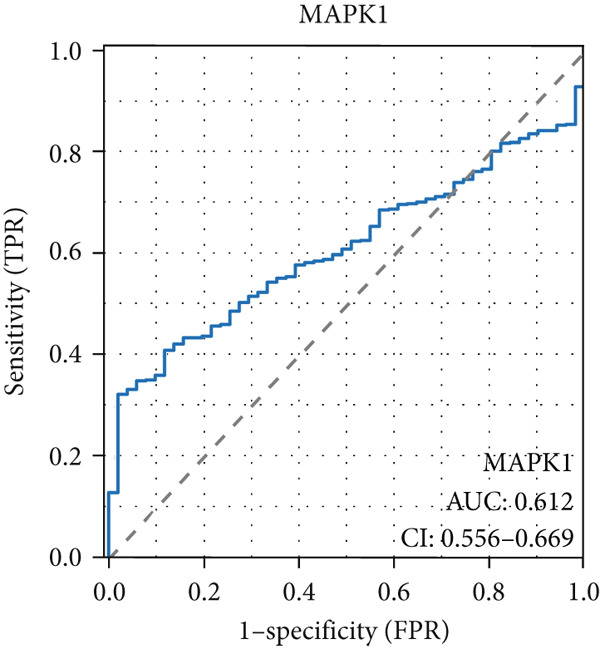
(d)

**Figure:**
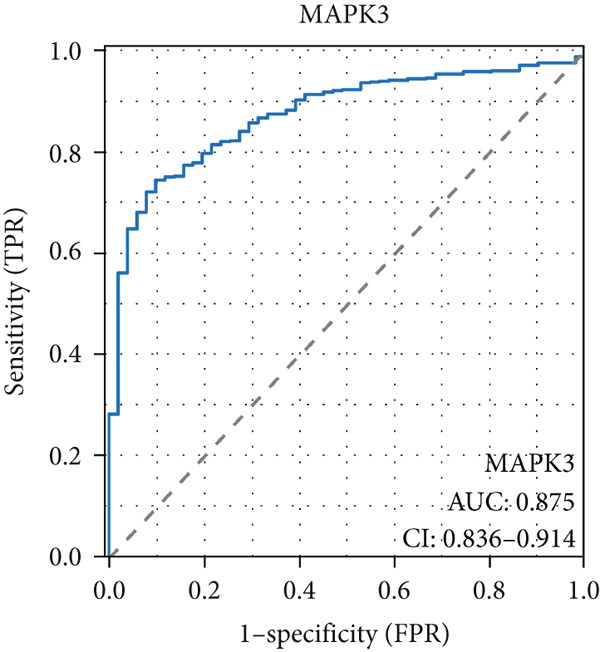
(e)

### 3.5. The Prognostic Value of Hub Genes

The mRNA expression data from TCGA cohort was utilized to classify CRC patients into high and low expression groups according to the expression levels of each hub gene (divided by the mean value). As illustrated in Figure 6a, no significant difference in OS was observed between patients with high AKT1 expression and those with low AKT1 expression (HR 1.24, CI 0.87–1.78, *p* = 0.241). However, patients exhibiting high AKT1 expression showed obviously worse PFS compared to those with low AKT1 expression (HR 1.67, CI 1.18–2.35, *p* = 0.003, Figure 6a). Kaplan–Meier curves indicated there were no significant differences in OS (HR 1.40, CI 0.96–2.03, *p* = 0.078) and PFS (HR 1.31, CI 0.96–1.78, *p* = 0.093) between patients with different ESR1 expression (Figure 6b). Similarly, no significant difference in OS (HR 0.80, CI 0.56–1.14, *p* = 0.212) and PFS (HR 0.87, CI 0.63–1.19, *p* = 0.369) was observed between patients with high and low JUN expression (Figure 6c). The patients with high MAPK1 expression exhibited a better OS than those with low MAPK1 expression (HR 0.62, CI 0.44–0.88, *p* = 0.008), while there was no significant difference in PFS (HR 0.80, CI 0.58–1.10, *p* = 0.176, Figure 6d). Furthermore, while no significant difference in OS was observed between patients with high and low expression of MAPK3 (HR 1.40, CI 0.99–1.98, *p* = 0.060), those with high expression of MAPK3 had a worse PFS (HR 1.44, CI 1.04–2.00, *p* = 0.027, Figure 6e).

Figure 6Kaplan–Meier survival analysis of five hub genes in TCGA cohort. (a) Kaplan–Meier survival analysis of OS (up) and PFS (down) between patients with high AKT1 expression and those with low AKT1 expression. (b) Kaplan–Meier survival analysis of OS (up) and PFS (down) between patients with high ESR1 expression and those with low ESR1 expression. (c) Kaplan–Meier survival analysis of OS (up) and PFS (down) between patients with high JUN expression and those with low JUN expression. (d) Kaplan–Meier survival analysis of OS (up) and PFS (down) between patients with high MAPK1 expression and those with low MAPK1 expression. (e) Kaplan–Meier survival analysis of OS (up) and PFS (down) between patients with high MAPK3 expression and those with low MAPK3 expression.

**Figure:**
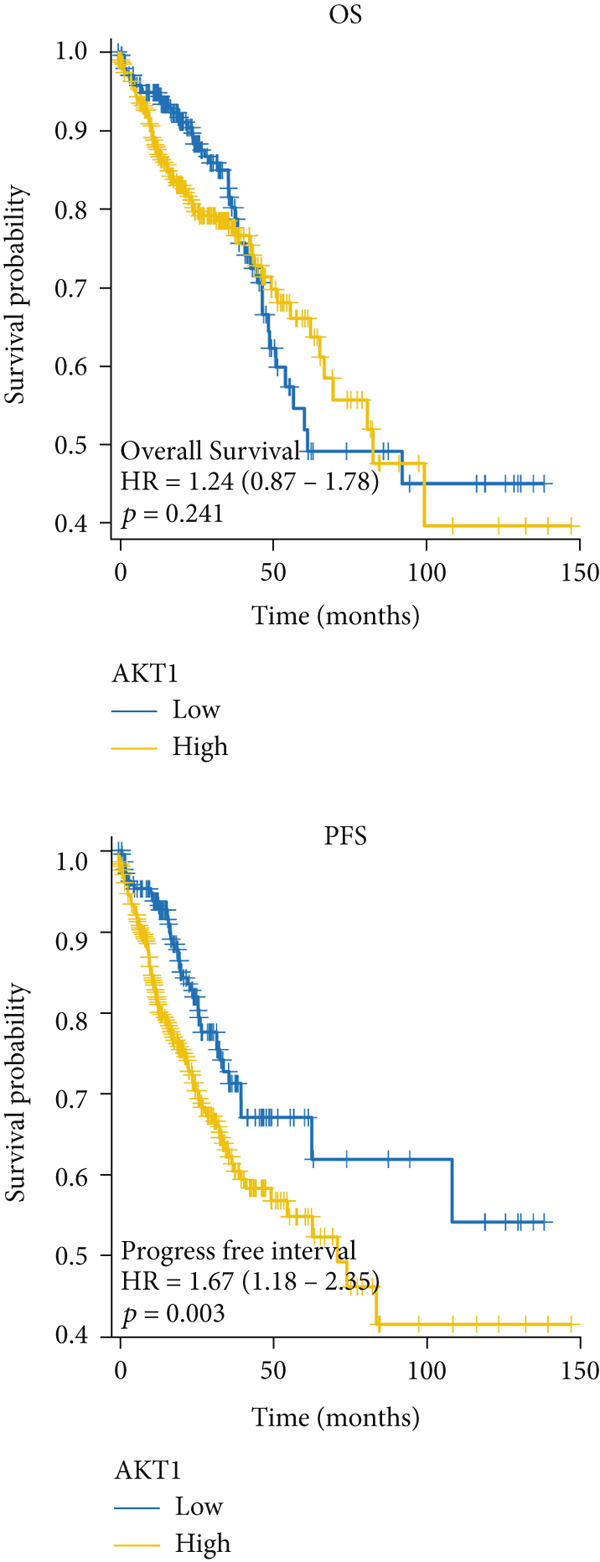
(a)

**Figure:**
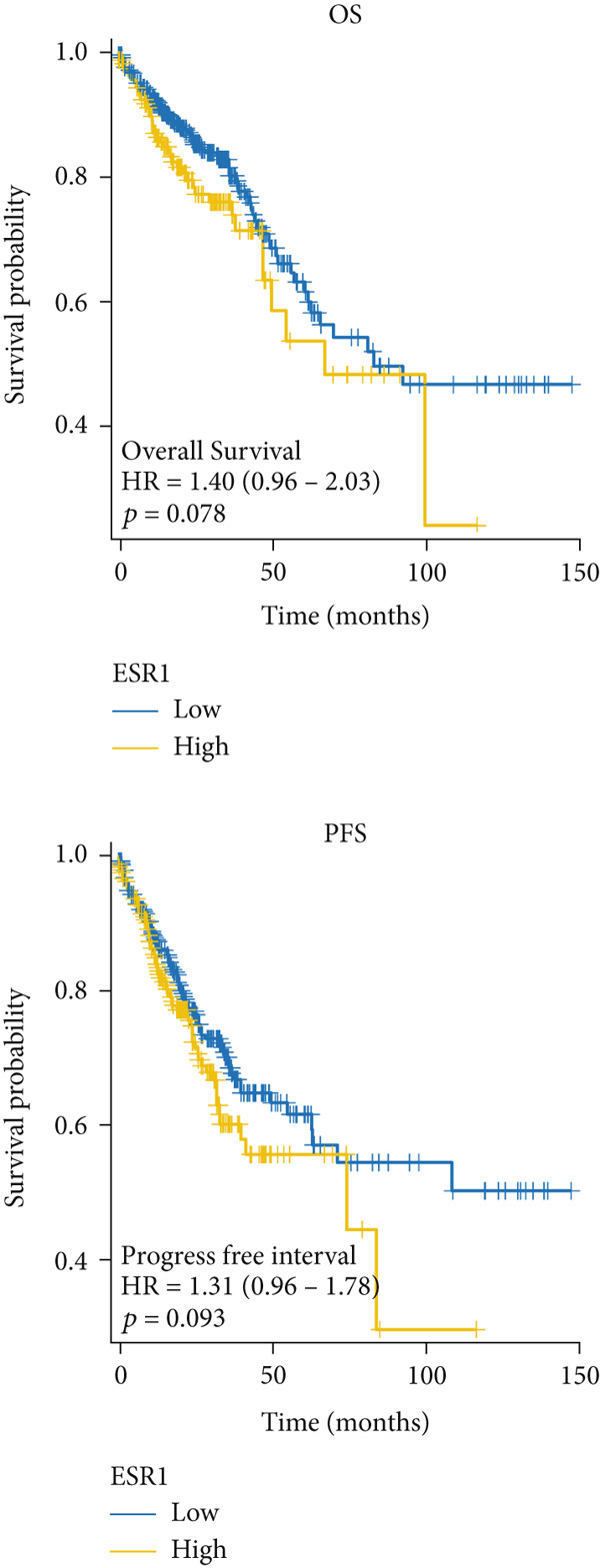
(b)

**Figure:**
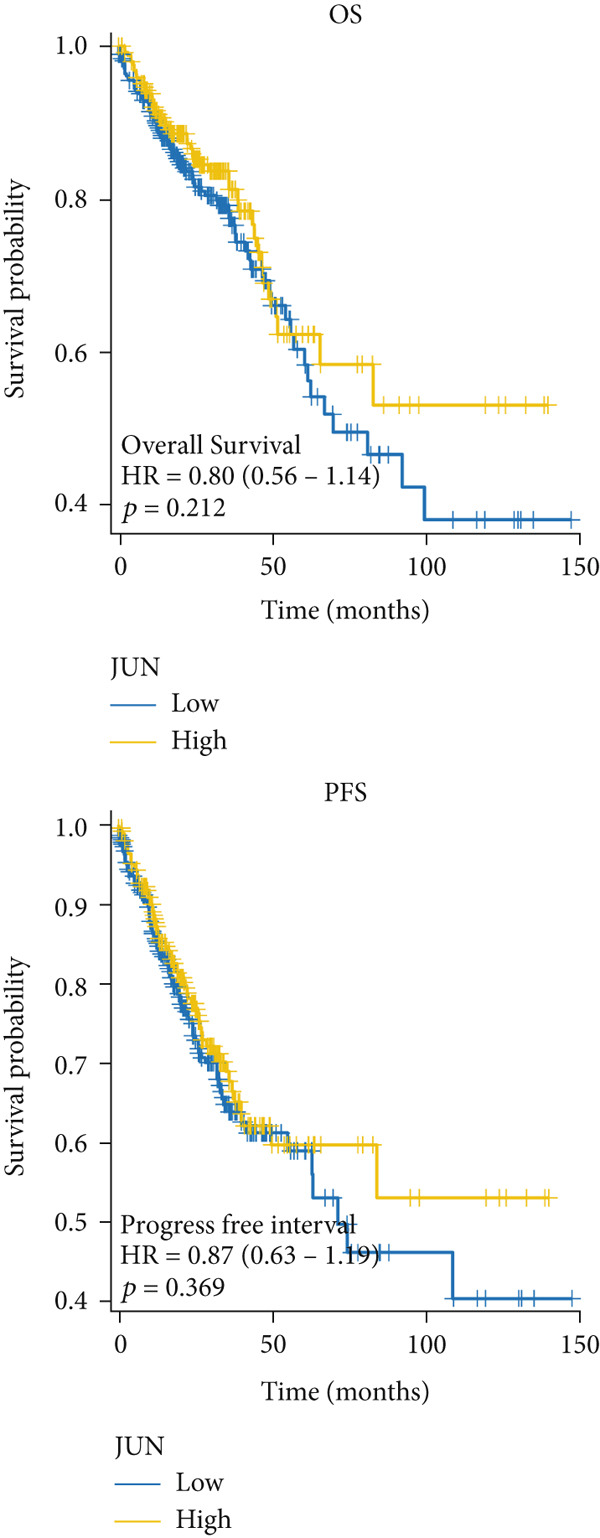
(c)

**Figure:**
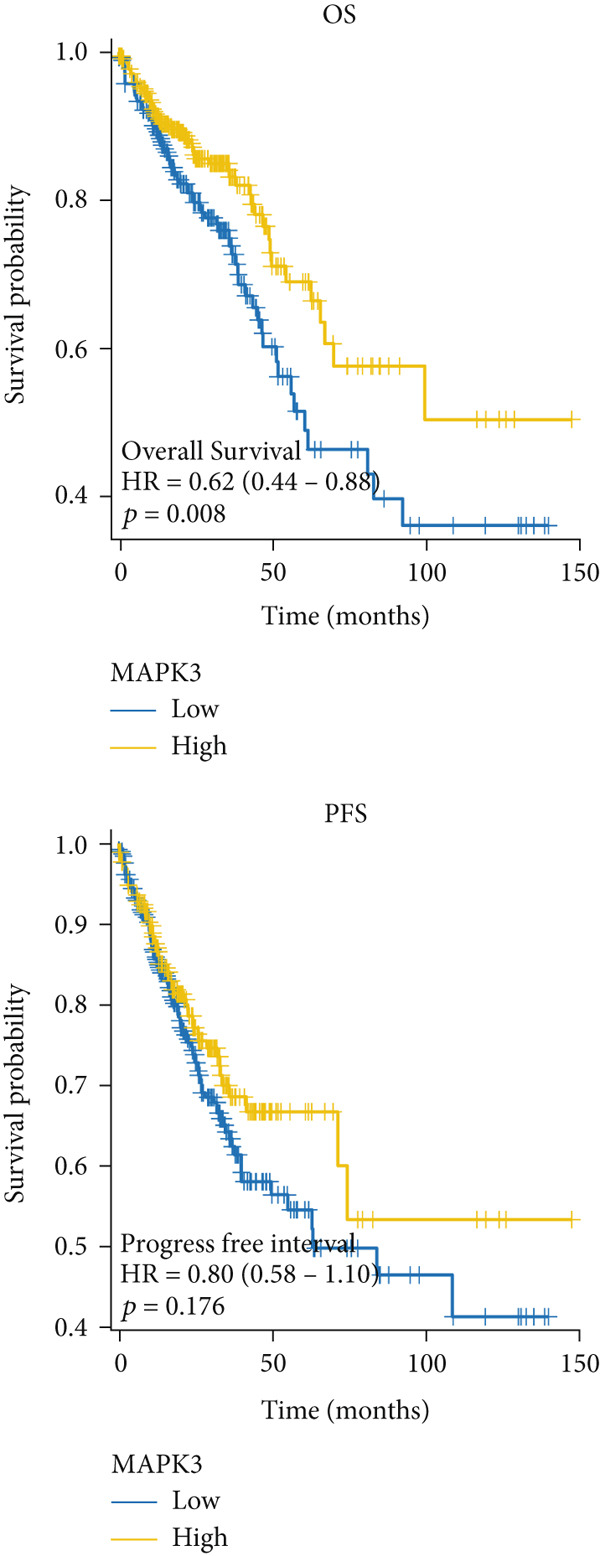
(d)

**Figure:**
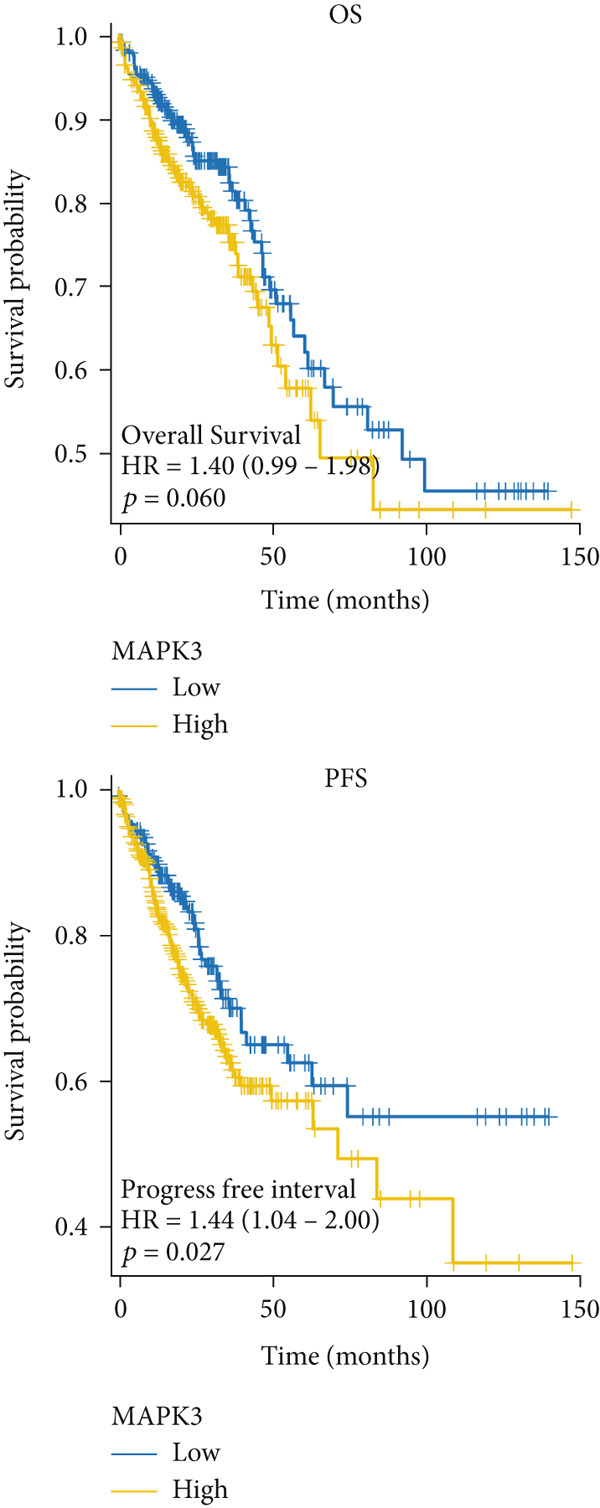
(e)

### 3.6. Verification the Distribution of Hub Genes in Tumor Microenvironment

To investigate the expression patterns of five key hub genes in CRC, single‐cell RNA sequencing datasets (GSE234804 and GSE277669) from CRC were analyzed. After preprocessing GSE234804 data based on the quality control and filtration, 8161 cells remained for further analysis (Figure 7a). Figure 7b illustrated the original identity of each cell. Subsequently, we visualized the high‐dimensional single‐cell RNA sequencing data and successfully classified cells into five subclusters (Figure 7c), including endothelial cells, monocytes, B cells, epithelial cells, and tissue stem cells. Finally, we found that MAPK1, MAPK3, AKT1, and JUN were distributed across different cell types of CRC tissues (Figure 7d).

Figure 7Single‐cell RNA sequencing analysis of GSE234804 cohort. (a) Quality control. (b) UMAP visualization of the original identity of each cell. (c) UMAP visualization of cell clusters. (d) Distribution of hub genes in cell clusters.

**Figure:**
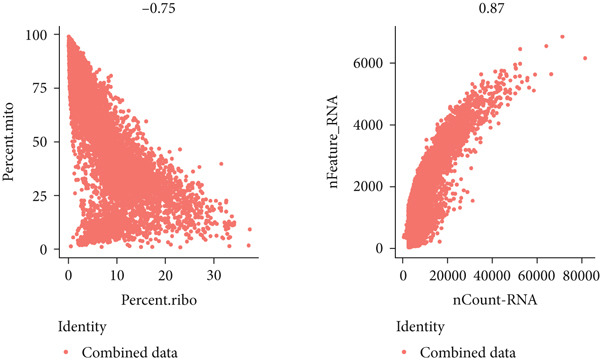
(a)

**Figure:**
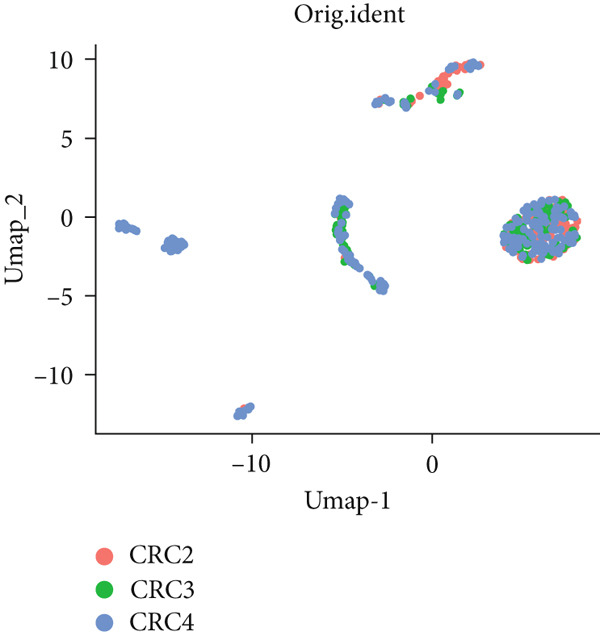
(b)

**Figure:**
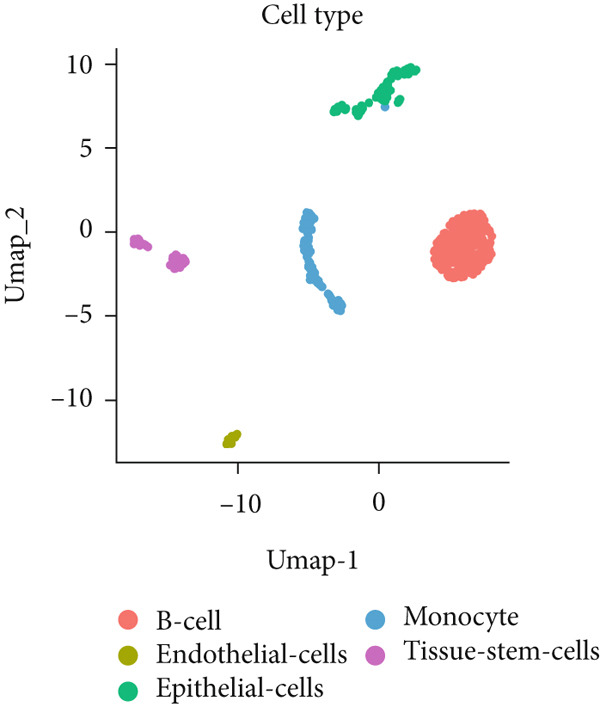
(c)

**Figure:**
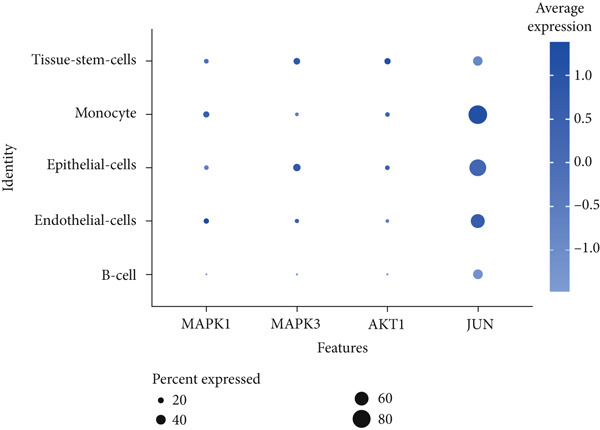
(d)

Similarly, after quality control and filtration for the single‐cell RNA sequencing data from GSE277669, 1502 high‐quality cells were screened and illustrated in Figure 8a,b. Subsequently, the major identified cell types included B cells, epithelial cells, CD8+ T‐cells, macrophages, fibroblasts, and monocytes (Figure 8c). MAPK1, MAPK3, AKT1, ESR1, and JUN were all distributed in these cell clusters, and JUN was highly expressed in the six cell subtypes (Figure 8d).

Figure 8Single‐cell RNA sequencing analysis of the GSE277669 cohort. (a) Quality control. (b) UMAP visualization of the original identity of each cell. (c) UMAP visualization of cell clusters. (d) Distribution of hub genes in cell clusters.

**Figure:**
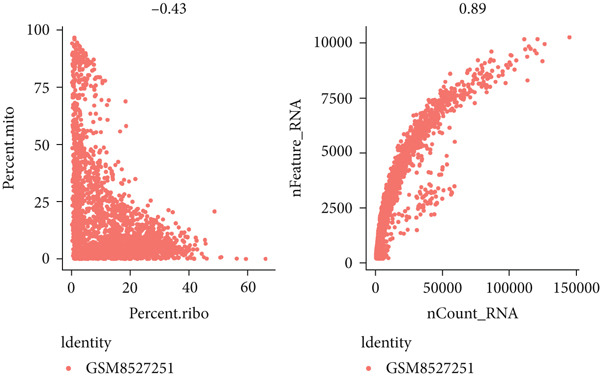
(a)

**Figure:**
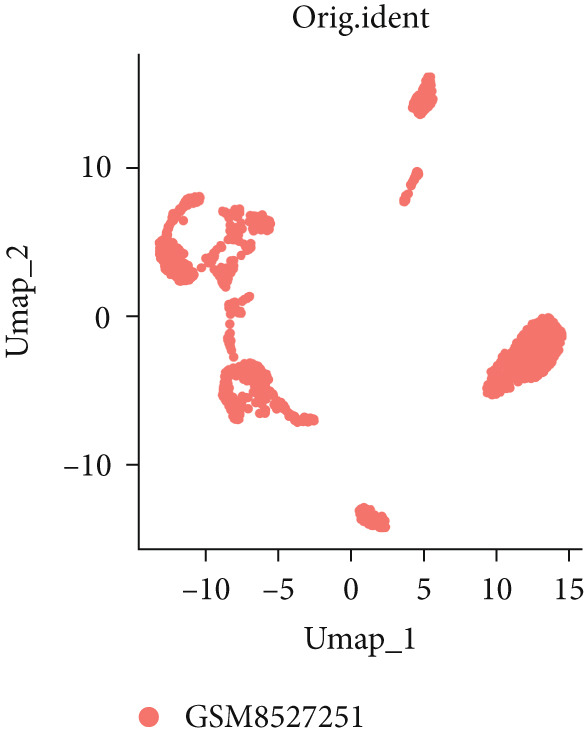
(b)

**Figure:**
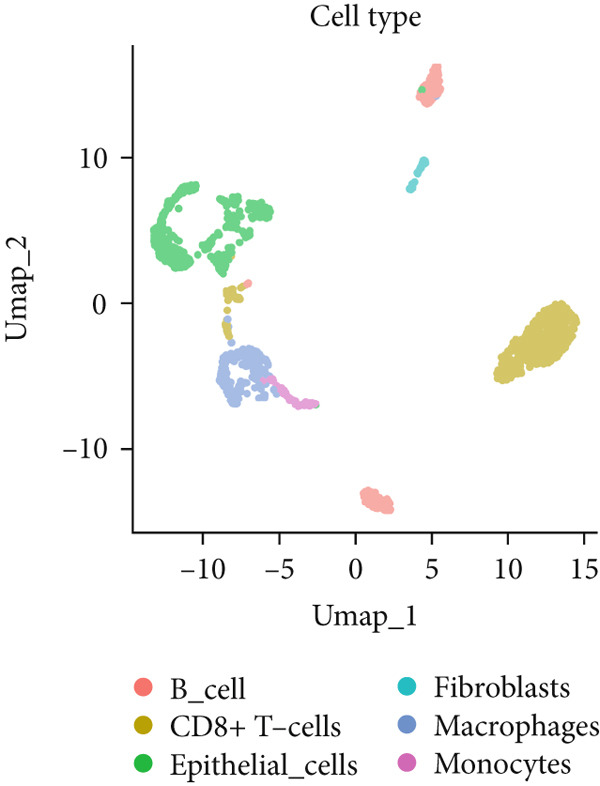
(c)

**Figure:**
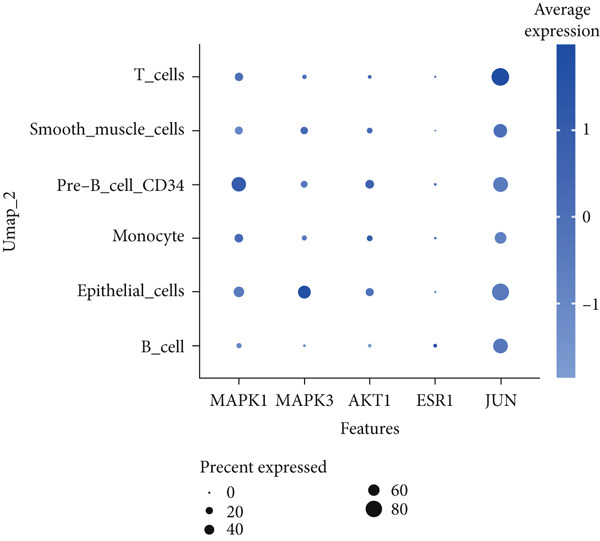
(d)

### 3.7. H_2_S Inhibited Cell Proliferation and Promoted Cell Apoptosis

Here, we used NaHS (an exogenous H_2_S donor) to mimic the role of H_2_S gas in CRC cells. Following treatment with 0.1–100 *μ*M NaHS for 24 h, the viability of HT‐29 cells and HCT‐116 cells was significantly inhibited compared to the DMSO group (*p* < 0.05, Figure 9a,b). Subsequently, TUNEL staining was conducted on cells treated with NaHS under a concentration of 5 or 10 *μ*M. The results showed that both concentrations of NaHS enhanced cell apoptosis of CRC compared to the DMSO group (*p* < 0.05, Figure 9c,d). Finally, the colony‐formation assay showed that HT‐29 cells and HCT‐116 cells treated with both concentrations of NaHS significantly inhibited colony formation compared to the DMSO group (Figure 9e,f). Taken together, these results confirmed that exogenous H_2_S had an inhibitory impact on the proliferation of CRC.

Figure 9H_2_S inhibited cell proliferation and promoted apoptosis for CRC. Cell viability detection of (a) HT‐29 and (b) HCT‐116 cells under treatment of NaHS with different concentrations by CCK‐8 kit. TUNEL staining of (c) HT‐29 and (d) HCT‐116 cells under treatment of NaHS. Colony formation assay of (e) HT‐29 and (f) HCT‐116 cells under treatment of NaHS. “ns” indicated no significance;  ^∗∗^ *p* < 0.01 and  ^∗∗∗^ *p* < 0.001. The incubation concentration was 5 *μ*M for HT‐29 cells and 10 *μ*M for HCT‐116 cells.

**Figure:**
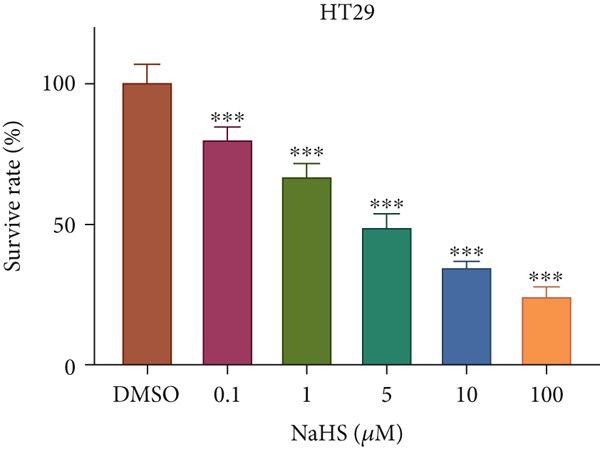
(a)

**Figure:**
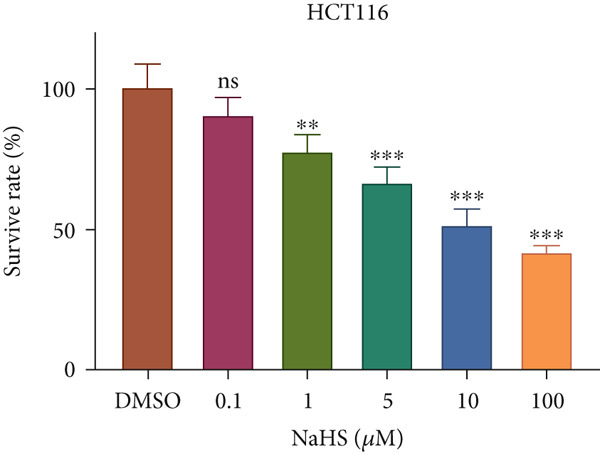
(b)

**Figure:**
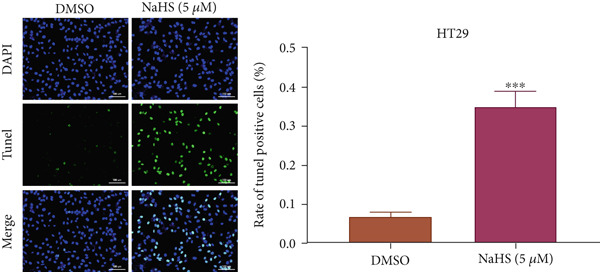
(c)

**Figure:**
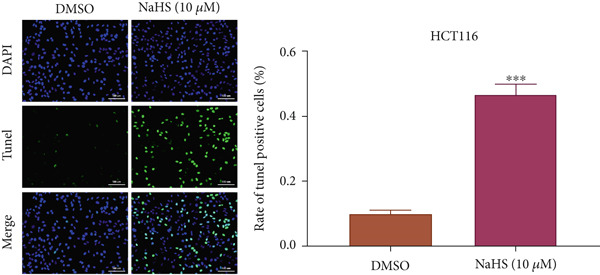
(d)

**Figure:**
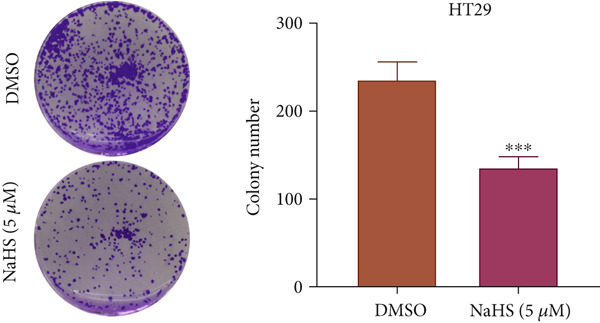
(e)

**Figure:**
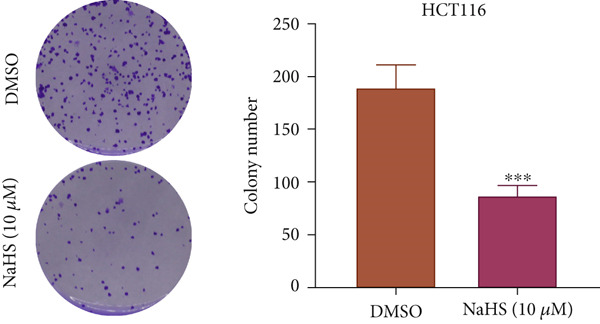
(f)

### 3.8. Effect of H_2_S on Hub Genes

Next, we conducted q‐PCR experiments to investigate whether H_2_S suppresses CRC by modulating the expression of hub genes. Compared with the DMSO group, both concentrations for NaHS observably inhibited the expression of AKT1, JUN, MAPK1, and MAPK3 in HT‐29 and HCT‐116 cells (*p* < 0.05, Figure 10a,b). However, the mRNA expression of ESR1 was not significantly changed in NaHS‐treated cells compared with DMSO (*p* > 0.05, Figure 10a,b). Taken together, H_2_S may inhibit cell growth in CRC by decreasing the mRNA levels of AKT1, JUN, MAPK1, and MAPK3.

Figure 10The mRNA expression of hub genes in NaHS‐treated CRC cells. (a) The mRNA expression of hub genes in 5 *μ*M NaHS‐treated HT‐29 cells. (b) The mRNA expression of hub genes in 10 *μ*M NaHS‐treated HCT‐116. “ns” indicates no significance;  ^∗^ *p* < 0.05 and  ^∗∗∗^ *p* < 0.001.

**Figure:**
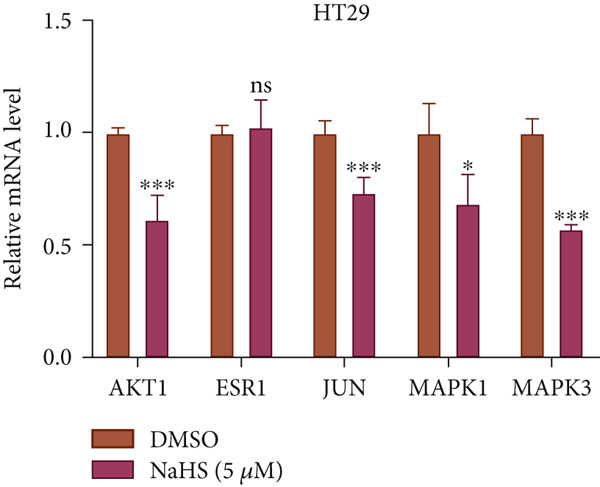
(a)

**Figure:**
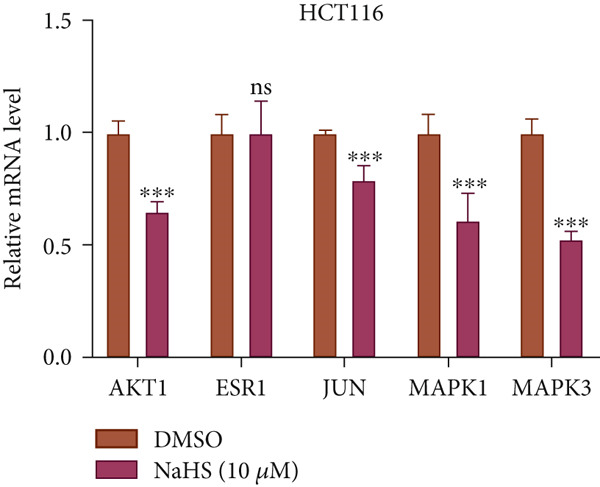
(b)

## 4. Discussion

CRC is a highly heterogeneous malignant tumor that threatens human life worldwide [33]. Despite significant progress in the early detection and management of CRC, both the incidence and mortality rates of this disease remain on the rise [34]. Therefore, seeking new therapeutic techniques and targets is crucial for improving the management of CRC. H_2_S is an endogenous bioactive gas that exerts important regulatory functions in multiple pathophysiological processes linked to various types of tumors, including colon cancer and lung cancer [35]. The level of H_2_S in the CRC organization is significantly elevated, and reducing the level of H_2_S can enhance the effect of immunotherapy [36]. Moreover, Wu et al. [37] found that H_2_S could be used as a novel treatment for cancer. For instance, H_2_S donor GYY4137 could inhibit the proliferation of Caco‐2 cells (human CRC cells) by inducing cell cycle arrest and cell death [38]. Therefore, further research on the role of H_2_S in CRC will contribute to the development of more effective diagnostic and therapeutic methods for CRC. Here, a combined approach of multiomics analysis with experimental verification was employed for the function of exogenous H_2_S in CRC and identifying its potential target.

Here, we identified 322 potential targets closely related to H_2_S treatment of CRC through multiomics analysis and multiple biomedical databases. Then, leveraging the network interaction modeling capability, we demonstrated the complex interaction among these targets and extracted five key hub genes: MAPK1, MAPK3, AKT1, ESR1, and JUN.

MAPK1 and MAPK3, also known as extracellular regulated kinase (ERK2 and ERK1), function in several cell signaling cascades and regulate cellular processes [39]. MAPK1, an essential oncogene, has been shown to be a key target for drug or small molecule therapy in CRC [40, 41]. In addition, it has been suggested that blocking the ERK1/2 signaling pathway can rescue cell proliferation, with therapeutic implications in CRC [42]. Besides, the promotion of oral cancer cell proliferation by H_2_S is associated with the activation of ERK1/2 [43]. AKT1 encodes a serine/threonine kinase that maintains genomic stability by participating in DNA replication and cell cycle regulation [44]. N‐Acetylmuramic acid was found to inhibit the proliferation of CRC by inhibiting the activation of AKT1 [45]. Additionally, H_2_S stimulated angiogenesis, involving the activation of AKT1 [46]. Furthermore, Liu et al. [47] reported that ESR1 is a potential drug target of genistein against CRC. However, the relationship between ESR1 and H_2_S has not been reported. JUN functions as cell signal transduction and gene expression regulation [48]. Lu et al. [49] have found that improving the abnormal expression of JUN (an important member of the AP‐1 transcription factor family) can inhibit the progression of CRC. Earlier research has validated that H_2_S exerts an influence on the activity of AP‐1 [50]. The above information indicates that the regulatory effects of H_2_S on CRC involve the engagement of multiple genes.

Besides, our results found ESR1 and MAPK3 had good diagnostic value for CRC (AUC > 0.8), while MAPK1, AKT1, and MAPK3 could predict the prognosis of CRC. This information indicated that MAPK3 has good diagnostic and prognostic value for CRC. MAPK3, also known as ERK1, has been found to be closely related to the formation of vasculogenic mimicry in CRC [51]. Meantime, research suggested that the activation of the MAPK‐Erk1/2 signaling pathway is associated with H_2_S concentration [52]. The above results indicated that MAPK3 is an important target for H_2_S in regulating CRC. Additionally, our findings demonstrated that H_2_S may exhibit anti‐CRC effects by modulating key biological functions, such as cellular response to chemical stress and the IL‐17 signaling pathway. Research reported that the activation of the IL‐17 signaling pathway affects tumorigenesis in CRC [53]. These above results indicated that H_2_S demonstrates anti‐CRC influence through the modulation of multiple targets and pathways.

Single‐cell RNA sequencing enables the analysis of examining gene expression at the level of individual cells [54], which provides support for understanding the distribution of key hub genes in the CRC tumor microenvironment. Specifically, GSE234804 and GSE277669 datasets revealed JUN had high expression in the immune cell population. Reports indicated that the activity of AP‐1 family members (Jun and Fos) is regulated by multiple immune signaling pathways [55]. These findings suggested that JUN may be involved in the immune regulation of the CRC tumor microenvironment.

H_2_S has emerged as a potential target in cancer treatment [7]. An earlier study found that the H_2_S donor GYY4137 could suppress cell proliferation of CRC by affecting both the cell cycle and cell death [38]. Similarly, we found that NaHS, another most commonly used H_2_S donor, inhibited cell proliferation and promoted cell apoptosis in CRC. These findings demonstrated that exogenous H_2_S may inhibit CRC growth. Besides, NaHS significantly reduced the mRNA expressions of MAPK1, MAPK3, AKT1, and JUN in CRC cells. Wei et al. [40] showed that the downregulation of AKT1 and MAPK1 expression can inhibit proliferation in CRC. In addition, decreased MAPK3 expression is associated with ferroptosis in CRC cells [56]. The tumor growth of CRC is closely related to the phosphorylation of JUN [57]. Collectively, the results demonstrated that H_2_S might suppress CRC proliferation through the downregulation of MAPK1, MAPK3, AKT1, and JUN.

There may be some possible limitations in this study. First, the screened dataset is relatively small, and our findings need to be validated in more datasets. Secondly, due to the retrospective nature of data obtained from public databases, some data may be incomplete or missing. Thirdly, further in vitro and in vivo studies are needed to validate the molecular mechanisms underlying the anti‐CRC effects of H_2_S.

## 5. Conclusion

In summary, through multiomics analysis, we discovered the potential five targets of H_2_S in regulating CRC, namely, MAPK1, MAPK3, AKT1, ESR1, and JUN. Meantime, the regulatory effects of H_2_S on CRC may be linked to the cellular response to chemical stress as well as the IL‐17 signaling pathway. Furthermore, MAPK3 has good diagnostic and prognostic performance for CRC. Exogenous H_2_S inhibited cell proliferation and downregulated the expression of MAPK1, MAPK3, AKT1, and JUN in CRC. In conclusion, our research brings novel perspectives on the therapeutic application of H_2_S in CRC.

## Disclosure

All authors consent to the publication of this study.

## Conflicts of Interest

The authors declare no conflicts of interest.

## Author Contributions

F.C. and C.Y.: conception and design. W.Z. and J.S.: data analysis and interpretation. Y.L. and F.L.: manuscript writing.

## Funding

No funding was received for this manuscript.

## Data Availability

The data that support the findings of this study are available from the corresponding author upon reasonable request.
